# Deep-learning-based 3D content-based image retrieval system on chest HRCT: Performance assessment for interstitial lung diseases and usual interstitial pneumonia

**DOI:** 10.1016/j.ejro.2025.100670

**Published:** 2025-07-23

**Authors:** Akira Oosawa, Atsuko Kurosaki, Atsushi Miyamoto, Shigeo Hanada, Yuichiro Nei, Hiroshi Nakahama, Yui Takahashi, Takahiro Mitsumura, Hisashi Takaya, Tomohisa Baba, Tae Iwasawa, Masatoshi Hori, Shoji Kido, Takashi Ogura, Noriyuki Tomiyama, Kazuma Kishi, Meiyo Tamaoka

**Affiliations:** a Medical Systems Research & Development Center, FUJIFILM Corporation, Minato-ku, Tokyo, Japan; b Department of Diagnostic Radiology, Fukujuji Hospital, Kiyose, Tokyo, Japan; c Department of Respiratory Medicine, Respiratory Center, Toranomon Hospital, Minato-ku, Tokyo, Japan; d Department of Respiratory Medicine, Toranomon Hospital, Kawasaki, Kanagawa, Japan; e Department of Respiratory Medicine, Kanagawa Cardiovascular and Respiratory Center, Yokohama, Kanagawa, Japan; f Department of Radiology, Kanagawa Cardiovascular and Respiratory Center, Yokohama, Kanagawa, Japan; g Department of Artificial Intelligence in Diagnostic Radiology, Osaka University Graduate School of Medicine, Suita, Osaka, Japan; h Diagnostic and Interventional Radiology, Osaka University Graduate School of Medicine, Suita, Osaka, Japan; i Division of Respiratory Medicine, Department of Internal Medicine, Toho University Graduate School of Medicine, Ota-ku, Tokyo, Japan

**Keywords:** Chest HRCT, Content-based image retrieval system, Deep learning, Interstitial lung diseases, Usual interstitial pneumonia

## Abstract

**Background:**

Diffuse parenchymal lung diseases have various conditions and CT imaging findings. Differentiating interstitial lung diseases (ILDs) and determining the presence or absence of usual interstitial pneumonia (UIP), can be challenging, even for experienced radiologists. To address this challenge, we developed a 3D-content-based image retrieval system (CBIR) and investigated its clinical usefulness.

**Methods:**

Using deep learning technology, we developed a prototype system that analyzes thin-slice whole lung HRCT images, automatically registers them in a database, and retrieves similar images. To evaluate search performance, we used a database of 2058 cases and assessed image similarity between query and retrieved cases using a 5-point visual score (5: Similar, 4: Somewhat similar, 3: Neither, 2: Somewhat dissimilar, 1: Dissimilar). To assess clinical usefulness, we evaluated the concordance of labels (ILD/non-ILD, with/without UIP) between query and retrieved cases, using a database of 301 cases across 57 diseases.

**Results:**

For search performance, the mean score of visual similarity between 70 queries and their top 5 retrieved cases was 4.37 ± 0.83. For clinical usefulness, label concordance between 25 queries and their top 5 retrieved cases was assessed across 4 labels. For ILD, the mean concordance of labels was 0.94 ± 0.15, while for non-ILD, it was 0.64 ± 0.31. For cases with UIP, the mean concordance of labels was 0.86 ± 0.17, while for cases without UIP, it was 0.83 ± 0.24.

**Conclusions:**

Our CBIR system showed high accuracy for identifying cases with/without UIP, suggesting its potential to support UIP differentiation in clinical practice.

## Highlights

• Developed a fully automated 3D-CBIR system using deep learning technology.
• The system showed high accuracy for identifying cases with/without UIP.
• The system may support the differential diagnosis of UIP in clinical practice.

## 1 Introduction

Diffuse parenchymal lung diseases (DPLDs), such as interstitial lung diseases (ILDs), are common in general hospitals but are often difficult to diagnose due to the wide variety of disease types and CT imaging findings [1]. Even among experienced radiologists, high interobserver variability in identifying CT findings has been reported [2], [3], [4].

To address this issue, image diagnosis support systems using artificial intelligence (AI)--whose performance has been dramatically improved by deep learning (DL) technology--have been applied in recent years. Walsh et al. investigated the use of DL technology for the automated classification of fibrotic lung diseases in high-resolution CT (HRCT) images, based on the criteria specified in international diagnostic guidelines [5], [6], and demonstrated its potential to achieve human-level accuracy [7]. Furukawa et al. developed an algorithm that combines DL and machine learning to differentiate Idiopathic pulmonary fibrosis (IPF) from other ILDs, achieving an average diagnostic accuracy of 83.6 % [8]. Furthermore, two biomarkers, developed using machine learning technology to quantify fibrosis, had been approved by FDA in 2024 and are now used in clinical practice [9], [10]. However, the process of AI classification and quantification remains a black box, making interpretation difficult [11], [12].

One system that has been extensively researched as a diagnostic support tool is the content-based image retrieval (CBIR) system, which uses a different approach to disease classification and biomarkers [13]. The CBIR system searches a database for previous cases with similar image findings and supports differential diagnosis of a new cases by referencing the diagnostic results of similar cases [14], [15], [23]. Because CBIR displays similar CT images rather than classification results or biomarkers, users can intuitively identify similar CT images and select the necessary information. We believe this avoids the problem of a black box.

CBIR applied to DPLDs has traditionally been a region of interest (ROI)-based system that manually encloses and searches lesion areas [14], [15], [16], with limited systems analyzing the entire lung [17]. However, recent advances in DL technology and faster processing speeds now enable high-precision quantitative analysis of whole lung HRCT images for DPLDs [18], [19], [20], [21]. Using the results of quantitative analysis of whole lung HRCT images, the development of 3D-CBIR systems is progressing, and their clinical usefulness is being evaluated [22], [23], [24]. In ROI-based search, the ROI of the lesion area must be manually enclosed and registered in the database [14], [15]. However, the technology in which AI automatically analyzes whole lung HRCT images eliminates the need for manual registration and enables fully automated retrieval [22]. Therefore, this technology is expected to be rapidly adopted in clinical practice in the future. Although few studies have reported on the clinical usefulness of CBIR applied to DPLDs, research using ROI-based retrieval systems [14], [15], [16] has shown that referencing similar cases can improve differential diagnosis. In another study using whole lung CT images for retrieval [23], a database of 288 patients with findings of usual interstitial pneumonia (UIP), nonspecific interstitial pneumonia (NSIP), cryptogenic organizing pneumonia (COP), and chronic hypersensitivity pneumonitis (CHP) was evaluated. The study aimed to investigate how the differential diagnosis of the four conditions varied when similar cases were referenced during a reading experiment, compared to when they were not. It found that referencing similar cases improved the differential diagnosis of UIP and NSIP. However, the types of findings that AI can identify with CBIR used in previous studies are limited, and few studies have explored specific scenarios where CBIR support is effective in the differential diagnosis flow. In this study, we developed a prototype 3D-CBIR system with fully automated database registration and retrieval, utilizing artificial intelligence-based quantitative CT image analysis software (AIQCT) [19]. AIQCT uses DL technology to analyze thin-slice whole lung HRCT images and classify voxels in the lung field into 28 different findings. The prototype system was applied to the case database, and its clinical usefulness for differential diagnosis was evaluated.

## 2 Materials and methods

### 2.1 The prototype system

#### 2.1.1 Prototype system

We developed a prototype system that automatically analyzes new whole lung HRCT images upon input through background processing. This system calculates the similarity between input images and existing cases in the database, then registers the input images to the database. Users can search for similar cases by simply selecting any image from the case list, displaying it, and pressing the search button on the screen. Similar images retrieved from the database are displayed in order of similarity.

#### 2.1.2 Definition of similarity and feature values

This system uses lung field regions and image feature values, obtained from AIQCT technology developed in a previous study [19] to calculate the similarity between query images and cases registered in the database. As detailed in [19], AIQCT technology includes four AI-based segmenters: lung extraction, airway extraction, pulmonary vessel extraction, and lung parenchyma segmentation. A three-dimensional U-Net was used to develop the first three segmenters. A two-dimensional convolutional-neural network was used to develop lung parenchyma segmentation. For lung parenchyma segmentation, all images were normalized to 0.5 × 0.5 × 0.5 mm isotropic voxels, and 32 × 32-pixel regions of interest (ROI) were extracted as input data. For the similarity calculation, the segmentation results of the lung field regions from AIQCT [19] were used to divide the lung field into 33 regions across 4 levels: whole lung (1 region), left/right lungs (2 regions), upper/middle/lower * left/right (6 regions), upper/middle/lower * dorsal/ventral * inner/outer * left/right (24 regions), based on the specific distribution of the image findings [1], [5], [25]. Similarity was calculated for each region. These regions were divided based on volume ratios: upper, middle, and lower at 1:1:1; dorsal and ventral at 1:1; and inner and outer at 1:2. The weighted sum of the similarities across these 33 regions was then used to determine the final similarity. The feature values used to calculate the similarity were the percentage values for 26 of the 28 findings from AIQCT analysis [19], as well as other features such as the mean and variance of the CT values, volume, and lesion percentage. Each feature value was normalized using the mean and deviation of the feature values from all case images registered in the database.

The granularity of the feature values varied across the 9 regions (whole lung, left/right lungs, upper/middle/lower * left/right) and the 24 regions (upper/middle/lower * dorsal/ventral * inner/outer * left/right). To classify image findings more coarsely for wider regions and in greater detail for local regions, similarity was calculated using a set of 12 feature values for the 9 regions and a more detailed set of 30 feature values for the 24 regions.

**Fig. 1:**
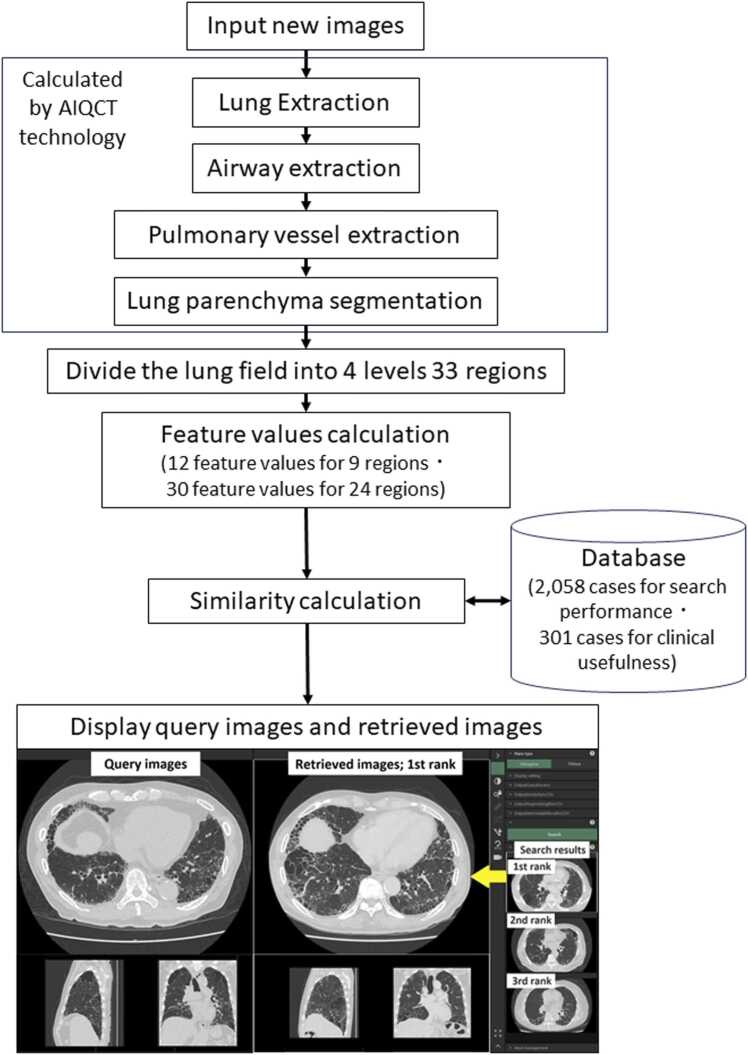
Flowchart of image processing in our prototype system. When new images are input, this system uses AIQCT technology to calculate the similarity between the input images and the existing cases in the database. After displaying the query image and clicking on the search button, the thumbnail images will then be displayed in order of search ranking (1st, 2nd, 3rd, etc.). Double clicking a thumbnail image in the search results will display the query image and the selected retrieved image side by side. In the capture screen of the prototype system in this figure, the 1st ranked images are displayed side by side.

Fig. 1 shows the flowchart of the image processing in this system. Table 1 a) and b) show the breakdown of the feature values used, while Table 2 a) through e) show the definition of similarity.

**Table 1:** List of feature values used.

a) For 9 regions: whole lung (1 region), left/right lungs (2 regions), upper/middle/lower * left/right (6 regions)	
**12 feature values**	
(1) ∼ (11) Percentage in the region	(1) Consolidation + Pleural effusion + Pleural thickening
	(2) Ground-glass opacity + Centrilobular Ground-glass opacity
	(3) Tree-in-bud appearance + Small nodules (not centrilobular) + Centrilobular nodules
	(4) Interlobular septal thickening
	(5) Hyperlucency + Centrilobular emphysema + Panlobular emphysema
	(6) Cyst
	(7) Cavity surrounded by infiltration + Cavity surrounded by mass
	(8) Bronchial wall thickening + Bronchiectasis + Air bronchogram + Traction bronchiectasis
	(9) Normal lungs + Borderline between normal and hyperlucency + Faint ground-glass opacity + Borderline dilatation of bronchioles
	(10) Honeycombing
	(11) Reticulation + Fine reticulation
(12) Volume	

b) For 24 regions: top/middle/bottom * dorsal/ventral * inside/outside * left/right	
30 feature values	
(1) ∼ (26) Percentage in the region	(1) Consolidation
	(2) Pleural effusion
	(3) Pleural thickening
	(4) Ground-glass opacity
	(5) Centrilobular Ground-glass opacity
	(6) Tree-in-bud appearance
	(7) Small nodules (not centrilobular)
	(8) Centrilobular nodules
	(9) Interlobular septal thickening
	(10) Hyperlucency
	(11) Centrilobular emphysema
	(12) Panlobular emphysema
	(13) Cyst
	(14) Cavity surrounded by infiltration
	(15) Cavity surrounded by mass
	(16) Bronchial wall thickening
	(17) Bronchiectasis
	(18) Air bronchogram
	(19) Traction bronchiectasis
	(20) Normal lung
	(21) Borderline between normal and hyperlucency
	(22) Faint ground-glass opacity
	(23) Borderline dilatation of bronchioles
	(24) Honeycombing
	(25) Reticulation
	(26) Fine reticulation
(27) Volume	
(28) Mean of CT values in the region	
(29) Variance of CT values in the region	
(30) Percentage of lesions in the region (sum of 24 findings, excluding normal lung and borderline between normal and hyperlucency)

**Table 2:** Definition of similarity.

a) Similarity of whole lung; S0
$S0=\sum_{m=1}^{M0}{\mathrm{wr}0}_{m}\left[\mathrm{SQRT}\left\{\sum_{n=1}^{N0}{\mathrm{wf}0}_{m,n}{\left({\mathrm{fc}0}_{m,n}-{\mathrm{fq}0}_{m,n}\right)}^{2}\right\}\right]$ where, M0 = 1, N0 = 12, fc0: feature values of images in the target database, fq0: feature values of the query image, wr0: weight for area, wf0: weights for feature values
**b) Similarity of left/right lung; S1**
$S1=\sum_{m=1}^{M1}{\mathrm{wr}1}_{m}\left[\mathrm{SQRT}\left\{\sum_{n=1}^{N1}{\mathrm{wf}1}_{m,n}{\left({\mathrm{fc}1}_{m,n}-{\mathrm{fq}1}_{m,n}\right)}^{2}\right\}\right]$ where, M1 = 2, N1 = 12, fc1: feature values of images in the target database, fq1: feature values of the query image, wr1: weights for area, wf1: weights for feature values
c) Similarity of top/middle/bottom * left/right; S2
$S2＝\sum_{m=1}^{M2}{\mathrm{wr}2}_{m}\left[\mathrm{SQRT}\left\{\sum_{n=1}^{N2}{\mathrm{wf}2}_{m,n}{\left({\mathrm{fc}2}_{m,n}-{\mathrm{fq}2}_{m,n}\right)}^{2}\right\}\right]$ where, M2 = 6, N2 = 12, fc2: feature values of images in the target database, fq2: feature values of the query image, wr2: weights for area, wf2: weights for feature values
d) Similarity of upper/middle/bottom * dorsal/ventral * inner/outer * left/right; S3
$S3＝\sum_{m=1}^{M3}{\mathrm{wr}3a}_{m}\left[\mathrm{SQRT}\left\{\sum_{n=1}^{N3a}{\mathrm{wf}3}_{m,n}{\left({\mathrm{fc}3}_{m,n}-{\mathrm{fq}3}_{m,n}\right)}^{2}\right\}\right]+\sum_{m=1}^{M3}{\mathrm{wr}3b}_{m}\left[\mathrm{SQRT}\left\{\sum_{n=N3a+1}^{N3b}{\left({\mathrm{fc}3}_{m,n}-{\mathrm{fq}3}_{m,n}\right)}^{2}\right\}\right]$ where, M3 = 24, N3a= 26, N3b= 30, fc3: feature values of images in the target database, fq3: feature values of the query image, wr3a, wr3b: weights for area, wf3: weights for feature values
**e) Total similarity; S**_**total**_
$S_{\mathrm{total}}=\mathrm{ws}0\times S0+\mathrm{ws}1\times S1+\mathrm{ws}2\times S2+\mathrm{ws}3\times S3$ where, ws0, ws1, ws2, ws3: weights for each similarity

Weights for area (wr0, wr1, wr2, wr3a, wr3b) are set according to the proportion of lesion (findings other than normal lung and borderline between normal and hyperlucency) in each area of the query image, using the following formula. w*r*= 1/ (1 + exp (-5.0*(proportion of lesion −0.5))) In the query image, the area with a large proportion of lesion will contribute more to the similarity.

Weights for feature values (wf0, wf1, wf2, wf3) are set according to the proportion of each finding in each area of each case in the database, using the following formula. wf= 1.5/ (1 + exp (300.0*proportion of finding))+0.25

This increases the contribution to similarity for feature values of findings with a small proportion of existence. Weights for each similarity (ws0, ws1, ws2, ws3) were used to normalize the number of divided regions and the dimensionality of feature values, as the four similarities (s0, s1, s2, s3) contribute equally to the total similarity (S_total_).

### 2.2 Development of the case database

This study was conducted in accordance with the amended Declaration of Helsinki and approved by the Institutional Review Board of Toranomon Hospital (No. 1925), Kanagawa Cardiovascular and Respiratory Center (No. KCRC-18–0039), Osaka University (No. 19061), and Fujifilm Corporation (No. #162).

For the evaluation of the developed CBIR system, a total of 2058 whole lung HRCT test cases were collected from three hospitals: Toranomon Hospital (254 cases of DPLDs with clinical diagnosis, collected from the date of imaging between 2006 and 2022), Kanagawa Cardiovascular and Respiratory Center (47 cases of DPLDs with clinical diagnosis, collected between from the date of imaging 2005 and 2020), Osaka University (1757 cases without clinical diagnosis, collected blindly from the date of imaging between 2019 and 2020). All cases were obtained during routine clinical examinations.

Written informed consent was obtained for the data collected from Toranomon Hospital and Kanagawa Cardiovascular and Respiratory Center. For the data collected from Osaka University, the requirement for informed consent was waived due to its anonymous and retrospective nature.

The following two case databases were created for the evaluation. All cases registered in these databases were selected from data not used for AI training in the previous study [19].

#### 2.2.1 Database for the evaluation of search performance

The database was created using all 2058 cases. Table 3 a) shows the detail of 2058 cases.

**Table 3:** Details of cases registered in databases.

a) 2058 cases for the evaluation of search performance	
**Item**	**Breakdown**
Sex: number	Male: 1230, Female: 828
Age: (y)	67.1 ± 14.4
Image size (pix)	512
Slice thickness (mm): number	0.25: 15, 0.5: 678, 0.6: 5, 0.625: 385, 1.0: 791, 1.25: 184
Tube Voltage (kV): number	100: 7, 120: 1902, 130: 1, 135: 2, 140: 146
Spatial resolution (mm)	Min: 0.488, Max: 1.270, Ave: 0.679
Manufacturer: number	TOSHIBA: 1263, GE: 569, SIEMENS: 195, Canon Medical Systems: 31,

b) 301 cases for the evaluation of clinical usefulness	
**Item**	**Breakdown**
Sex: number	Male: 202, Female: 99
Age: (y)	65.5 ± 13.2
Image size (pix)	512
Slice thickness (mm): number	0.5: 3, 0.625: 1, 1.0: 297
Tube Voltage (kV): number	100: 3, 120: 296, 130: 1, 135: 1
Spatial resolution (mm)	Min: 0.488, Max: 0.782, Ave: 0.641
Manufacturer: number	TOSHIBA: 268, Canon Medical Systems: 31, SIEMENS: 1, GE: 1

#### 2.2.2 Database for the evaluation of clinical usefulness

In this evaluation, 301 cases across 57 diseases, obtained at Toranomon Hospital and Kanagawa Cardiovascular and Respiratory Disease Center, were used out of the 2058 cases used to evaluate the search performance. Clinical diagnoses were assigned to these 301 cases, and correct labels (ILD/non-ILD, with/without UIP) used for the evaluation were determined through agreement between a radiology specialist (A.K.) and a respiratory specialist (A.M.) with 44 and 25 years of experience, respectively. When determining the presence of UIP, any case classified as “Indeterminate for UIP” or higher was considered to have UIP. Details of the 301 cases are shown in Table 3 b). Clinical diagnoses and the number of correct labels for the 301 cases are shown in Table 4.

**Table 4:** Clinical diagnoses and the number of correct labels of 301 cases registered in the database for evaluating clinical usefulness^1^

**Clinical diagnosis**	**Number of cases**	**Number of correct labels**			
		**ILD**	**Non-** **ILD**	**With** **UIP**	**Without UIP**
Idiopathic pulmonary fibrosis	39	39		39	
Chronic obstructive pulmonary disease (including Pulmonary emphysema)	23		23		23
Combined pulmonary fibrosis and emphysema	19	19		17	2
Nontuberculous mycobacteriosis	17		17		17
Radiation pneumonitis	16	16			16
Dermatomyositis	11	11		1	10
Collagen lung disease；RA	10	9	1	7	3
Hypersensitivity pneumonitis f-HP (n = 6) Other than f-HP (n = 4)	10	10		7	3
Scleroderma	10	10			10
Unclassifiable interstitial pneumonia	10	10		7	3
Anti-ARS antibody syndrome	9	9			9
Pulmonary tuberculosis	8		8		8
Cryptogenic organizing pneumonia	7	7			7
NSIP (including IgG4-related lung disease)	7	7			7
Pleuroparenchymal fibroelastosis	7	7		5	2
Pulmonary aspergillosis	7		7		7
Pneumocystis pneumonia	6		6		6
Acute fibrinous and organizing pneumonia	5	5			5
Bronchiectasis	5		5		5
Pneumoconiosis	5	5			5
Sarcoidosis	5		5		5
Acute exacerbation of idiopathic pulmonary fibrosis	4	4		4	
ANCA associated vasculitis MPA, including suspected (n = 3) Other AAV (n = 1)	4	4		2	2
Drug-induced lung damage	4	4			4
Multicentric Castleman’s disease	4		4		4
Pulmonary alveolar proteinosis	4		4		4
Pulmonary lymphangiomyomatosis	4		4		4
Diffuse panbronchiolitis	3		3		3
Miliary tuberculosis	3		3		3
Sjögren syndrome	3	2	1		3
Asbestosis, Round atelectasis, Pleural plaque	2		2		2
Chronic eosinophilic pneumonia	2	2			2
Disseminated cryptococcosis	2		2		2
Human adjuvant disease	2		2		2
Pneumococcal pneumonia	2		2		2
Pulmonary coccidioidomycosis	2		2		2
Pulmonary sequestration	2		2		2
Allergic bronchopulmonary mycosis	1		1		1
Alveolar hemorrhage	1		1		1
Aspiration pneumonia	1		1		1
Bronchial asthma	1		1		1
COVID−19 pneumonia	1	1			1
Idiopathic interstitial pneumonias (non UIP)	1	1			1
Influenza bacillus pneumonia	1		1		1
Langerhans histiocytosis	1		1		1
Legionella pneumonia	1		1		1
Multifocal micronodular type II pulmonary cell hyperplasia	1		1		1
Pneumothorax	1		1		1
Polymyalgia rheumatica	1	1		1	
Pulmonary arteriovenous malformation	1		1		1
Pulmonary infarction	1		1		1
Pulmonary ossification	1		1		1
Smoking related ILD	1	1		1	
Staphylococcus aureus pneumonia	1		1		1
Swyer-James syndrome	1		1		1
**Total**	301	184	117	91	210

### 2.3 Evaluation methods

#### 2.3.1 Evaluation of search performance

To evaluate the search performance, the image similarity between the query and the 1st to 5th ranked retrieved cases was assessed. One case was selected as a query from the database of 2058 cases, and a search was performed against the remaining 2057 cases. For the query cases, 70 cases were manually selected from the data of Toranomon Hospital and Kanagawa Cardiovascular and Respiratory Disease Center to include a wide range of image findings. Table 5 shows the clinical diagnoses of the 70 selected query cases. This evaluation was performed by an engineering technician (A.O.) with over 20 years of experience in developing diagnostic support technologies for DPLDs. In this evaluation, the clinical diagnosis was made blindly. The evaluation was performed by visually assessing the concordance of the presence and distribution of 26 image findings (Table 1 b) (1) ∼ (26)) between the query and retrieved images and assigning a 5-point score (5: Similar, 4: Somewhat similar, 3: Neither 2: Somewhat dissimilar, 1: Dissimilar). Since physicians may assess the similarity of images not only based on the consistency of image findings but also on their own differential diagnosis, we decided that this evaluation should be conducted by an engineer.

**Table 5:** Clinical diagnoses of selected 70 query cases for evaluating search performance^2^

Clinical diagnosis	Number of cases
Idiopathic pulmonary fibrosis	13
Cryptogenic organizing pneumonia	6
Nontuberculous mycobacteriosis	5
Acute fibrinous and organizing pneumonia	3
Drug-induced lung damage	3
Microscopic polyangiitis (including suspected)	3
Pulmonary tuberculosis	3
Unclassifiable interstitial pneumonia	3
Chronic obstructive pulmonary disease	2
Combined pulmonary fibrosis and emphysema	2
Pleuroparenchymal fibroelastosis	2
Pneumocystis pneumonia	2
Pulmonary lymphangiomyomatosis	2
Acute exacerbation of idiopathic pulmonary fibrosis	1
Alveolar hemorrhage	1
Aspiration pneumonia	1
Bronchiectasis	1
COVID−19 pneumonia	1
Collagen lung disease；RA	1
Diffuse panbronchiolitis	1
Hypersensitivity pneumonitis (f-HP)	1
Hypersensitivity pneumonitis (other than f-HP)	1
Idiopathic interstitial pneumonias (non UIP)	1
Influenza bacillus pneumonia	1
Legionnaires’ pneumophila	1
Miliary tuberculosis	1
Multicentric Castleman’s disease	1
Pneumococcal pneumonia	1
Pneumothorax	1
Pulmonary aspergillosis	1
Pulmonary coccidioidosis	1
Radiation pneumonitis	1
Sarcoidosis	1
Smoking related ILD	1
**Total**	70

#### 2.3.2 Evaluation of clinical usefulness

Clinical usefulness was evaluated based on the clinical practice flow (ILD or non-ILD → with UIP or without UIP) to narrow down candidates for differential diagnosis. The concordance of the correct labels (ILD or non-ILD, with UIP or without UIP) was evaluated for the query case and the 1st to 5th ranked retrieved cases. Due to time constraints in clinical practice, it is assumed that only the top search results will be referred to, so we decided to evaluate the retrieved results up to the 5th place. For evaluation, one case was selected as the query from the database of 301 cases, and a search was performed against the remaining 300 cases. Twenty-five cases were manually selected as queries for each correct label (ILD, non-ILD, with UIP, without UIP) to include as many diseases as possible. Since a query case can have multiple correct labels, a total of 59 cases were selected. The breakdown of the selected query cases is shown in Table 6.

**Table 6:** Clinical diagnoses and correct labels of 59 query cases for evaluating clinical usefulness^3^

Clinical diagnosis	Number of cases	Number of correct labels			
		**ILD**	**Non-ILD**	**With UIP**	**Without UIP**
Idiopathic pulmonary fibrosis	13	8		12	
Nontuberculous mycobacteriosis	5		5		2
Cryptogenic organizing pneumonia	3	3			3
Pulmonary tuberculosis	3		3		1
Unclassifiable interstitial pneumonia	3	2		3	
Chronic obstructive pulmonary disease	2		2		1
Combined pulmonary fibrosis and emphysema	2	1		2	
Drug-induced lung damage	2	2			1
Pleuroparenchymal fibroelastosis	2	2		2	
Pulmonary lymphangiomyomatosis	2		2		1
Acute exacerbation of idiopathic pulmonary fibrosis	1	1		1	
Aspiration pneumonia	1		1		1
Bronchiectasis	1		1		1
COVID−19 pneumonia	1				1
Collagen lung disease；RA	1	1		1	
Diffuse panbronchiolitis	1		1		1
Hypersensitivity pneumonitis (f-HP)	1	1			1
Hypersensitivity pneumonitis (other than f-HP)	1	1		1	
Idiopathic interstitial pneumonias (non-UIP)	1	1		1	
Influenza bacillus pneumonia	1		1		1
Legionnaires’ pneumophila	1		1		1
Microscopic polyangiitis (including suspected)	1	1		1	
Miliary tuberculosis	1		1		1
Multicentric Castleman’s disease	1		1		1
Pneumococcal pneumonia	1		1		1
Pneumocystis pneumonia	1		1		1
Pneumothorax	1		1		1
Pulmonary aspergillosis	1		1		1
Pulmonary coccidioidosis	1		1		1
Radiation pneumonitis	1	1			1
Sarcoidosis	1		1		1
Smoking related ILD	1			1	
**Total**	59	25	25	25	25

## 3 Results

### 3.1 Evaluation of search performance

Regarding search performance, the mean and standard deviation of the visual 5-point score between the 1st to 5th ranked retrieved cases across the 70 queries were 4.37 ± 0.83 (maximum: 5.00, minimum: 2.80, 95 % confidence interval (CI): 4.29–4.46), indicating high image similarity. The mean and standard deviation of the visual 5-point scores for each of the four labels were as follows: 4.50 ± 0.78 (95 % CI: 4.40–4.61) for ILD, 4.17 ± 0.85 (95 % CI: 4.03–4.32) for non-ILD, 4.86 ± 0.37 (95 % CI: 4.80–4.93) for cases with UIP, and 4.09 ± 0.88 (95 % CI: 3.97–4.20) for cases without UIP. Supplementary figure 1 a), b), and c) show examples of search results when querying for a) multicentric Castleman’s disease, b) IPF, and c) Pulmonary lymphangiomyomatosis, which were used in this evaluation.

### 3.2 Evaluation of clinical usefulness

Table 7 a) shows a breakdown of the results obtained from the ILD/non-ILD queries. For ILD, the mean and standard deviation of the concordance of the correct labels between the 25 queries and the 1st to 5th ranked retrieved cases was 0.94 ± 0.15 (95 % CI: 0.88–1.00). For non-ILD, the mean and standard deviation of the concordance of the correct labels was 0.64 ± 0.31 (95 % CI: 0.51–0.77). The concordance of correct labels was statistically significantly higher in ILD compared to non-ILD (p < 0.001). The total accuracy for identifying ILD and non-ILD cases was 0.79 ± 0.28 (95 % CI: 0.71–0.87). Table 7 b) shows a breakdown of the results obtained from the queries of cases with/without UIP. For cases with UIP, the mean and standard deviation of the concordance of the correct labels between the 25 queries and the 1st to 5th ranked retrieved cases was 0.86 ± 0.17 (95 % CI: 0.79–0.93). For cases without UIP, the mean and standard deviation of the concordance of the correct labels was 0.83 ± 0.24 (95 % CI: 0.73–0.93). When comparing cases with and without UIP, no statistically significant difference was found in the concordance of correct labels (p = 0.681). The total accuracy for identifying cases with and without UIP was 0.84 ± 0.20 (95 % CI: 0.79–0.90). Due to differences in the definitions of UIP and the AI technology, direct comparisons with our results are difficult. However, in the previous study [7], when classifying cases as UIP or non-UIP, the sensitivity of the algorithm was 79.3 % and specificity was 90.1 %, while the median sensitivity of radiologists was 65.5 % and specificity was 94.1 %.

**Table 7:** Evaluation results of the clinical usefulness for the queries^4^

a) ILD/non-ILD								
Label of query	Number of cases out of 25 query cases						Concordance of the correct labels mean±deviation	Search performance: visual score mean±deviation
	**Number of same correct label** **in the Top 5 retrieved cases**							
	**5/5**	**4/5**	**3/5**	**2/5**	**1/5**	**0/5**		
ILD	21	2	1	1	0	0	0.94 ± 0.15 95 %CI:0.88–1.00	4.50 ± 0.78 95 %CI:4.40–4.61
Non-ILD	6	6	5	5	1	2	0.64 ± 0.31 95 %CI:0.51–0.77	4.17 ± 0.85 95 %CI:4.03–4.32
Total^※^^1^	27	8	6	6	1	2	0.79 ± 0.28 95 %CI:0.71–0.87	-

b) With/without UIP								
Label of query	Number of cases out of 25 query cases						Concordance of the correct labels mean±deviation	Search performance: visual score mean±deviation
	**Number of same correct label** **in the Top 5 retrieved cases**							
	**5/5**	**4/5**	**3/5**	**2/5**	**1/5**	**0/5**		
With UIP	12	9	3	1	0	0	0.86 ± 0.17 95 %CI:0.79–0.93	4.86 ± 0.37 95 %CI:4.80–4.93
Without UIP	12	9	2	1	0	1	0.83 ± 0.24 95 %CI:0.73–0.93	4.09 ± 0.88 95 %CI:3.97–4.20
Total^※^^2^	24	18	5	2	0	1	0.84 ± 0.20 95 %CI:0.79–0.90	-

c) Idiopathic pulmonary fibrosis (correct label: ILD and with UIP)								
**Label of query**	**Number of cases out of** **13 IPF query cases**						**Concordance of the correct labels** mean±deviation	**Search performance of IPF:** **visual score** mean±deviation
	**Number of same correct label** **in the top 5 retrieved cases**							
	**5/5**	**4/5**	**3/5**	**2/5**	**1/5**	**0/5**		
ILD	13	0	0	0	0	0	1.00 ± 0.00 95 %CI:1.00–1.00	4.83 ± 0.38 95 %CI:4.74–4.92
With UIP	5	7	1	0	0	0	0.86 ± 0.13 95 %CI:0.79–0.94	

ILD vs. Non-ILD: p < 0.001 (student’s *t*-test for concordance of the correct labels)

※1 Number of cases out of 50 query cases

With UIP vs. Without UIP: p = 0.681 (student’s *t*-test for concordance of the correct labels)

※2 Number of cases out of 50 query cases

## 4 Discussion

### 4.1 Differentiation of ILD/non-ILD

The concordance of the correct labels between the 25 queries and the 1st to 5th ranked retrieved cases was 0.94 ± 0.15 for ILD and 0.64 ± 0.31 for non-ILD, showing significantly lower concordance for non-ILD cases. Of the 301 cases registered in the database, 117 cases were non-ILD, indicating that non-ILD cases were fewer than ILD cases. In addition, the mean visual score for search performance in non-ILD was 4.02 ± 0.83, lower than the 4.50 ± 0.78 observed in ILD, which could have contributed to the difference in concordance.

In non-ILD, concordance was particularly low, with 0.0 (0/5) for tuberculosis and aspiration pneumonia and 0.2 (1/5) for pneumococcal pneumonia. Since tuberculosis shows a variety of image patterns and distributions, such as consolidation, cavities, ground-glass opacities, and small nodules [26], similar image patterns can also be seen in ILD cases [1], which may make differentiation between the two challenging. In addition, since only one case of aspiration pneumonia and two cases of pneumococcal pneumonia were included in the database, ILD cases may have been mixed in the top five retrieved cases for these queries.

### 4.2 Differentiation of cases with/without UIP

Of the 301 cases registered in the database, 91 were with UIP and 210 were without UIP. The number of cases with UIP was about two-fifths of those without UIP, which created a disadvantageous condition for search performance. However, the concordance of the correct labels between the 25 queries and the 1st to 5th ranked retrieved cases for cases with UIP was 0.86 ± 0.17, which was almost the same as 0.83 ± 0.24 for cases without UIP, showing no statistically significant difference. For cases with UIP, the mean visual score of search performance was higher with a smaller deviation (4.86 ± 0.37), indicating that high concordance was maintained even when the number of registered cases in the database was small.

Table 7 c) shows a breakdown of the evaluation results for IPF (correct label: ILD and with UIP), which was included in 13 of the 59 queries. The mean visual score for search performance was very high at 4.83 ± 0.38, and the concordance of the correct labels was also high, with 1.00 ± 0.00 for the ILD label and 0.86 ± 0.13 for the with UIP label. These results suggest that our 3D-CBIR system may contribute to supporting the differentiation of UIP in clinical practice.

In the previous study [22], the retrieval accuracy of the 3D-CBIR system was evaluated using a database of 492 cases across 3 diseases (UIP, NSIP, and COP), by assessing the concordance of disease names between the 60 queries and the 1st to 5th ranked retrieved cases. Although the evaluation methods differ, the retrieved performance for UIP (88.7 %) was high, indicating consistency with our system. On the other hand, for cases without UIP, the mean visual score for search performance (4.09 ± 0.88) drops to almost 4.0, but the concordance of the correct labels remains high (0.83 ± 0.24). Similar to non-ILD, search performance for cases without UIP is lower due to the diverse range of diseases with different image patterns. However, the larger number of cases registered in the database for without UIP (N = 210) may have mitigated the decrease in concordance. When using the CBIR system in clinical practice, it will be important to control the number of cases registered in the database as search targets.

### 4.3 Future directions

As mentioned above, our 3D-CBIR system enables fully automated registration and retrieval from a database. By integrating it with Picture Archiving and Communication System (PACS) or image interpretation reporting systems, it can be seamlessly used in the image interpretation workflow without imposing additional burdens on physicians. Furthermore, by linking it with electronic medical records, it will be expected to enable reference to past treatment outcomes of similar cases, thereby providing valuable insights for the current treatment of patients.

### 4.4 Limitation

There are several limitations to this study. For the evaluation of clinical usefulness, the number of cases registered in the database is small (N = 301), which may cause the distribution of diseases to differ from that in actual clinical practice. Among 57 diseases included in the database 26 had only one or two registered cases. The number of correct labels assigned to the registered cases in the database was also unbalanced (ILD; N = 184/non-ILD; N = 117, with UIP; N = 91/without UIP; N = 210). In the future, further validation will be required for diseases with a small number of cases in the database by adding more cases. Further validation will also be required by changing the balance of correct labels for registered cases. Also, the query cases used for evaluation were selected manually, so there was a possibility of potential bias.

In addition, to verify the clinical usefulness of our CBIR system in differentiating UIP, a reading experiment will be necessary to compare changes in differential diagnoses with and without the system.

## 5 Conclusion

The 3D-CBIR system developed in this study provides retrieval results showed high accuracy for identifying cases with and without UIP, suggesting its potential to support UIP differentiation in clinical practice. Furthermore, the concordance of correct labels between the queries and the retrieved cases is influenced by the number of registered cases in the database. Therefore, the number of registered cases should be controlled when the system is used in clinical practice.

## CRediT authorship contribution statement

**Akira Oosawa:** Conceptualization, Methodology, Software, Validation, Formal analysis, Data curation, Writing – original draft. **Atsuko Kurosaki:** Conceptualization, Methodology, Validation, Formal analysis, Data curation, Writing – original draft. **Atsushi Miyamoto:** Supervision, Conceptualization, Methodology, Formal analysis, Data Curation, Resources, Writing – original draft. **Meiyo Tamaoka:** Supervision, Conceptualization, Methodology, Formal analysis, Data Curation, Resources, Writing – original draft. **Shigeo Hanada:** Resources, Data curation, Writing – review & editing. **Yuichiro Nei:** Resources, Data curation, Writing – review & editing. **Hiroshi Nakahama:** Resources, Data curation, Writing – review & editing. **Yui Takahash:** Resources, Data curation, Writing – review & editing. **Takahiro Mitsumura:** Resources, Data curation, Writing – review & editing. **Hisashi Takaya:** Resources, Data curation, Writing – review & editing. **Kazuma Kishi:** Supervision, Resources, Data curation, Writing – review & editing. **Tomohisa Baba:** Resources, Data curation, Writing – review & editing. **Tae Iwasawa:** Resources, Data curation, Writing – review & editing. **Takashi Ogura:** Resources, Data curation, Writing – review & editing. **Masatoshi Hori:** Resources, Data curation, Writing – review & editing. **Shoji Kido:** Resources, Data curation, Writing – review & editing. **Noriyuki Tomiyama:** Resources, Data curation, Writing – review & editing.

## Informed Consent Statement

Written informed consent was obtained for data collected from Toranomon Hospital and Kanagawa Cardiovascular and Respiratory Center. For data collected from Osaka University, the requirement for informed consent was waived due to the anonymous and retrospective nature.

## Institutional Review Board Statement

This study was conducted in accordance with the amended Declaration of Helsinki. The Institutional Review Board of Toranomon Hospital (No. 1925), Kanagawa Cardiovascular and Respiratory Center (No. KCRC-18–0039), Osaka University (No. 19061), and Fujifilm Corporation (No. #162) approved this study.

## Funding statement

This work was supported by a research grant from FUJIFILM Corporation.

## Declaration of Generative AI and AI-assisted technologies in the writing process

During the preparation of this work Akira Oosawa used DeepL Translate for some parts of the manuscript to translate Japanese sentences into English. After using this tool, the authors reviewed and edited the content as needed. As such, they take full responsibility for the content of the publication.

## Declaration of Competing Interest

Akira Oosawa is an employee of FUJIFILM Corporation. Atsuko Kurosaki received consulting fees from FUJIFILM Corporation and KONICA MINOLTA Corporation. Meiyo Tamaoka, Atsushi Miyamoto, Shigeo Hanada, Yuichiro Nei, Hiroshi Nakahama, Yui Takahash, Takahiro Mitsumura and Hisashi Takaya received an institutional research grant from Fujifilm Corporation for this study. Kazuma Kishi received an institutional research grant from Fujifilm Corporation for this study, while he was affiliated with Toranomon Hospital. Tomohisa Baba and Takashi Ogura received an institutional grant from Fujifilm Corporation for this study. Tae Iwasawa received research grants from Canon Medical Systems and Ziosoft, Inc. Masatoshi Hori has Collaborative Research Agreements with Fujifilm Corporation for this study. Atsushi Miyamoto received an honorarium from Nippon Boehringer Ingelheim Co., Ltd. Takashi Ogura received honoraria from Japanese Boehringer Ingelheim, Shionogi & Co., Ltd., Astellas Pharma, Toray Industries, Eisai Pharma, and Taiho Pharm. Tomohisa Baba received honoraria from Nippon Boehringer Ingelheim Co., Ltd., Shionogi & Co., Ltd., Daiichi Sankyo Co., Ltd., AstraZeneca K.K., Merck Biopharma Japan, Taiho Pharmaceutical Co., Ltd., and Bristol Myers Squibb K.K. Tae Iwasawa received an honorarium from Boehringer Ingelheim. Noriyuki Tomiyama received honoraria for lecturing from FUJIFILM Medical Co., Ltd. Takashi Ogura received payments for Advisory Board from Bristol-Myers Squibb Company, Taiho Pharmaceutical Co., Ltd., and Japanese Boehringer Ingelheim. Tomohisa Baba received payments for expert testimony from Daiichi Sankyo Co., Ltd., AstraZeneca K.K., Merck Biopharma Japan, and AbbVie GK. Shoji Kido has no competing interests to disclose.

## Data Availability

The datasets used and/or analyzed during the current study are confidential and not publicly available to protect the privacy of the participants.

## Footnotes

Appendix A Supplementary data associated with this article can be found in the online version at doi:10.1016/j.ejro.2025.100670.

## Footnotes

1 Abbreviations: AAV, ANCA associated vasculitis: ANCA, anti-neutrophil cytoplasmic antibody: ARS, aminoacyl t–RNA synthetases: f-HP, fibrotic hypersensitivity pneumonitis: IgG4, Immunoglobulin G4: ILD, interstitial lung disease: MPA, Microscopic polyangiitis: NSIP, nonspecific interstitial pneumonia: RA, rheumatoid arthritis; UIP, Usual interstitial pneumonia.

2 Abbreviations: f-HP, fibrotic hypersensitivity pneumonitis: ILD, interstitial lung disease: RA, rheumatoid arthritis; UIP, Usual interstitial pneumonia.

3 Abbreviations: f-HP, fibrotic hypersensitivity pneumonitis: ILD, interstitial lung disease: RA, rheumatoid arthritis; UIP, Usual interstitial pneumonia.

4 Abbreviations: ILD, interstitial lung disease: UIP, Usual interstitial pneumonia.
