# Supplementary material for: Deep-learning-based 3D content-based image retrieval system on chest HRCT: Performance assessment for interstitial lung diseases and usual interstitial pneumonia
Source: Eur J Radiol Open. 2025 Jul 23;15:100670. doi: 10.1016/j.ejro.2025.100670 (PMC12309587; doi:10.1016/j.ejro.2025.100670)
Supplement: Supplementary file 1 — Supplementary material [file mmc1.docx]

**SUPPLEMENTARY DATA**

**[Figure Legends]**

**Supplementary Figure 1:** Examples of the search results.

　The labels overlaid on the images indicate the identified findings (light blue: ground-glass opacity, blue: reticulation, orange: honeycombing, dark yellow: cyst, cavity, hyperlucency and emphysema, magenta: consolidation, peach: interlobular septal thickening and small nodules, purple: blood vessels, green: bronchi).

a) Query: Multicentric Castleman's disease
b) Query: Idiopathic pulmonary fibrosis

c) Query: Pulmonary lymphangiomyomatosis
