# Supplementary figures and images for: Deep-learning-based 3D content-based image retrieval system on chest HRCT: Performance assessment for interstitial lung diseases and usual interstitial pneumonia
Source: Eur J Radiol Open. 2025 Jul 23;15:100670. doi: 10.1016/j.ejro.2025.100670 (PMC12309587; doi:10.1016/j.ejro.2025.100670)

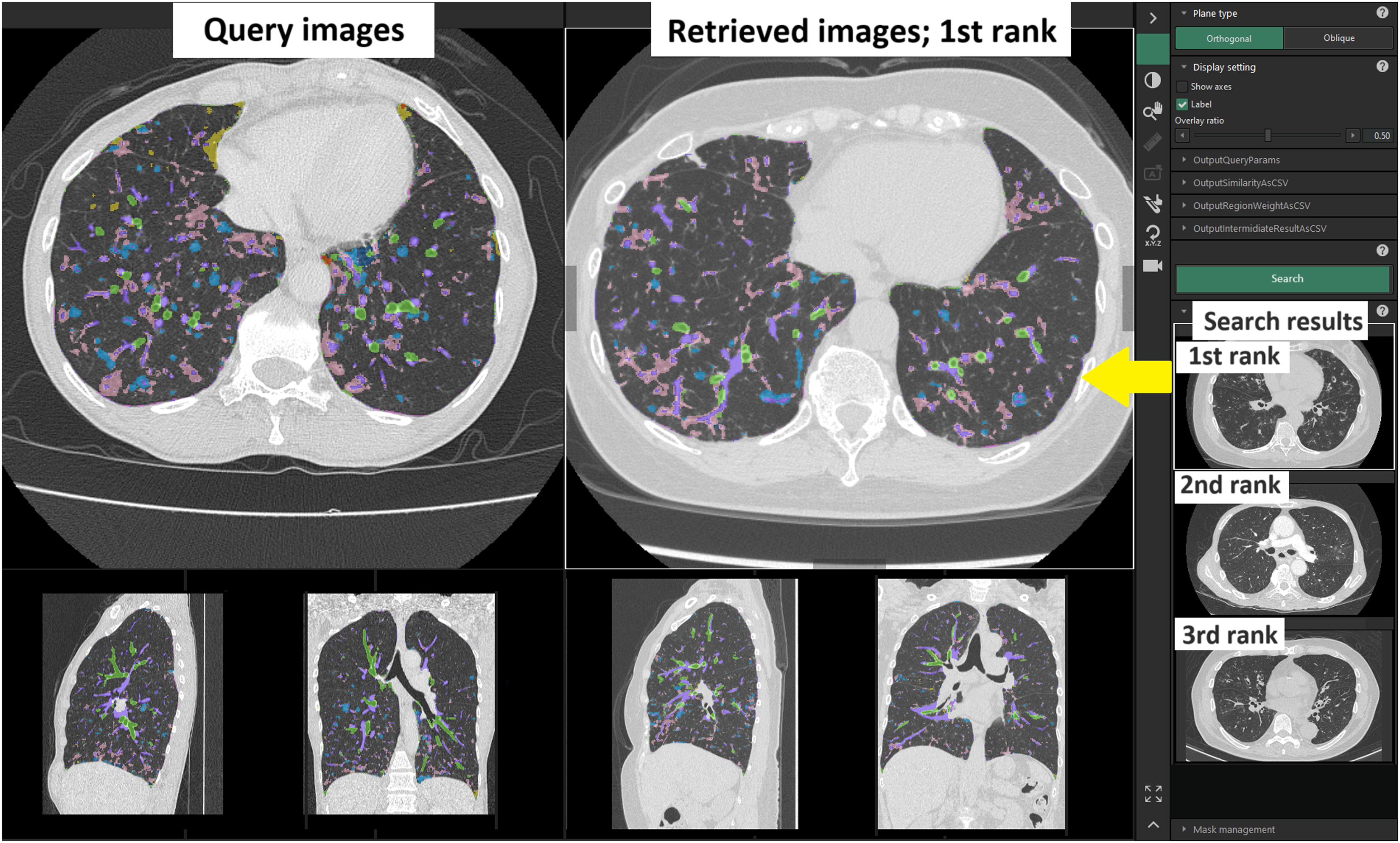

Supplement: Supplementary file 2 — Supplementary material [file mmc2.jpg]

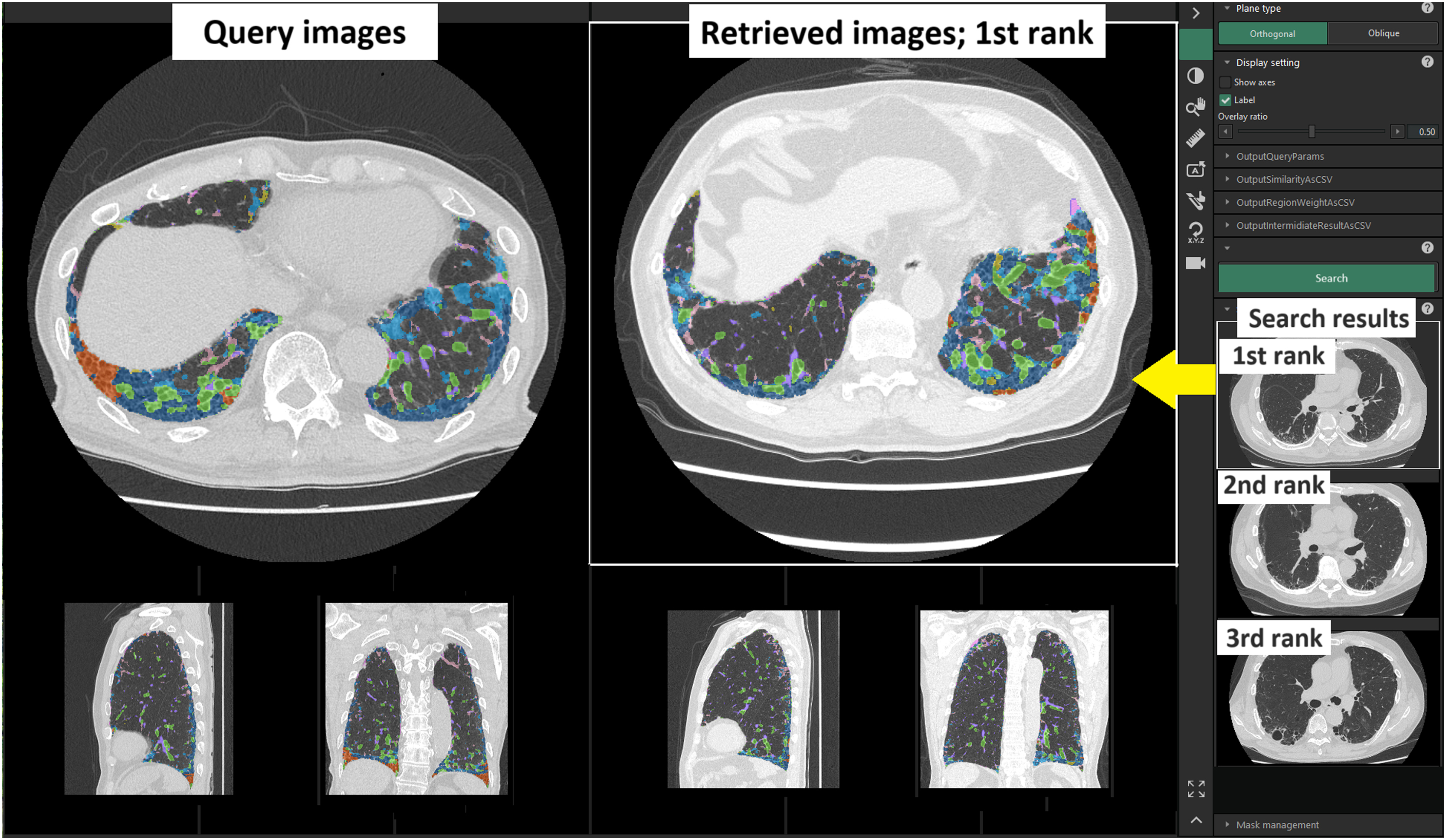

Supplement: Supplementary file 3 — Supplementary material [file mmc3.jpg]

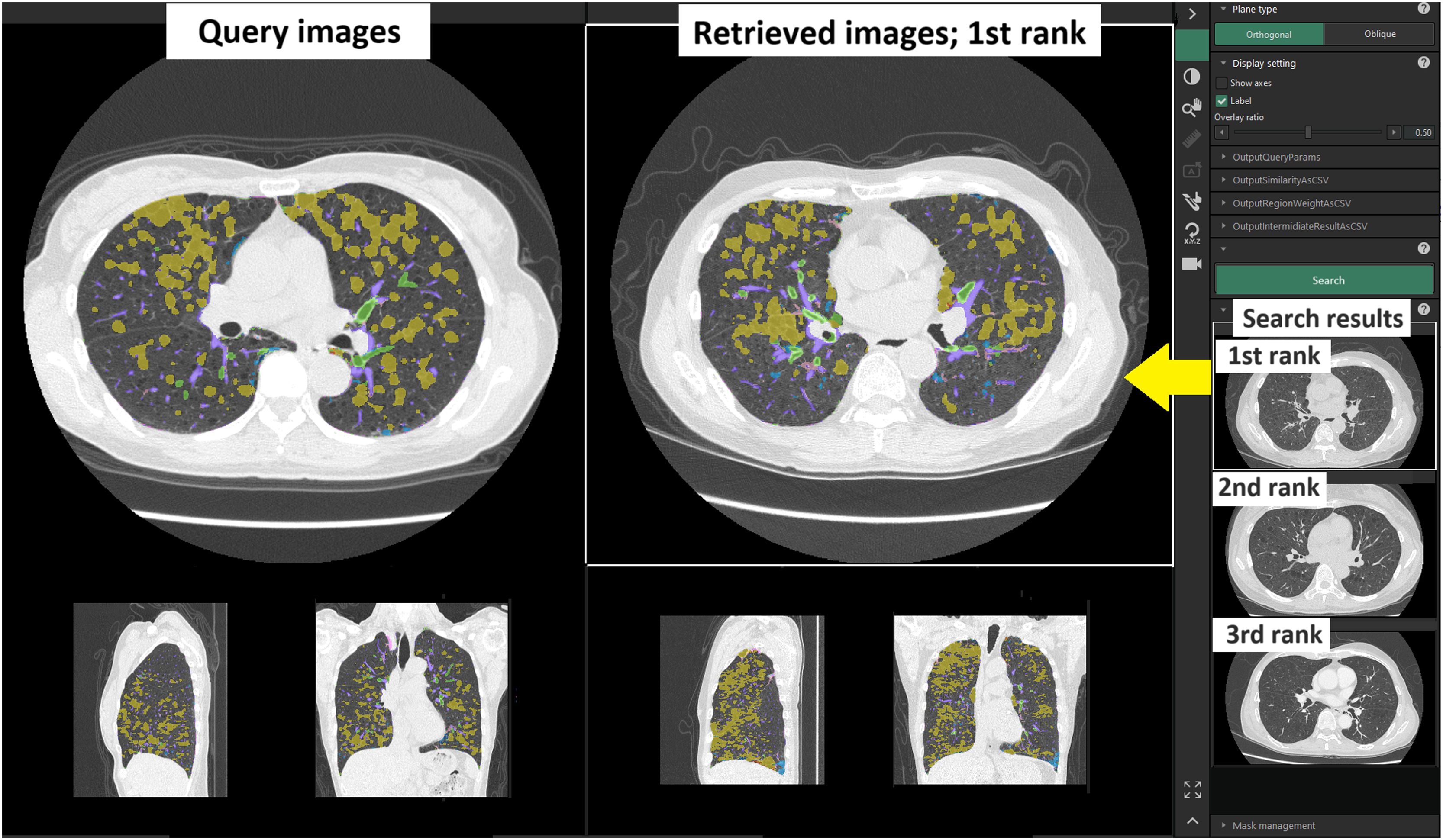

Supplement: Supplementary file 4 — Supplementary material [file mmc4.jpg]
